# Online Mental Health Survey in a Medical College in China During the COVID-19 Outbreak

**DOI:** 10.3389/fpsyt.2020.00459

**Published:** 2020-05-13

**Authors:** Jia Liu, Qing Zhu, Wenliang Fan, Joyman Makamure, Chuansheng Zheng, Jing Wang

**Affiliations:** ^1^ Department of Radiology, Union Hospital, Tongji Medical College, Huazhong University of Science and Technology, Wuhan, China; ^2^ Hubei Province Key Laboratory of Molecular Imaging, Wuhan, China; ^3^ Department of Neurology, Union Hospital, Tongji Medical College, Huazhong University of Science and Technology, Wuhan, China

**Keywords:** COVID-19, depression, anxiety, PHQ-9, GAD-7

## Abstract

**Background:**

The outbreak of coronavirus disease 2019 (COVID-19) in Wuhan, Hubei province, has led to the quarantine of many residents in their homes, in order to mitigate its spread. Some of these people developed mental health problems, and many solutions have been put in place to address the mental health issues of patients and health professionals affected by the disease. However, not much attention has been given to students, particularly those from medical school. The present study aims to conduct an online survey to investigate the mental health status of students from a medical college in Hubei province.

**Materials and Methods:**

The WeChat-based survey program Questionnaire Star, which contained questions from Patient Health Questionnaire-9 (PHQ-9) and Generalized Anxiety Disorder-7 (GAD-7), was utilized for the present study.

**Results:**

A total of 217 students participated in the survey. Among these students, 127 were female and 90 were male. Furthermore, 77 students (35.5%) who participated in the survey were in a state of depression, and 48 (22.1%) were in a state of anxiety. The majority of students who were in depressed (n=75) or anxiety (n=46) states had mild or moderate states. There were no significant differences in students in terms of gender, geographical location, and grade, for the prevalence of depression and anxiety.

**Conclusion:**

The present study implies that universities need to take measures to prevent, identify, and deal with mental health problems among students during large-scale stressors.

## Introduction

In December 2019, the outbreak of coronavirus disease 2019 (COVID-19) was reported in a wet market in Wuhan, Hubei province (1). The virus spread throughout China and extended worldwide (2), infecting more than 2,300,000 people, and resulted in more than 160,000 deaths as of April 20, 2020. Due to the rising numbers of confirmed cases and deaths, many people within and outside Hubei province were quarantined at home, including some of the college students from Hubei province. Many previous studies have indicated that stressful events can have an immense effect on an individual's psychological and physical well-being (3, 4). The outbreak of infectious diseases, such as severe acute respiratory syndrome (SARS), Middle East respiratory syndrome (MERS), and COVID-19, and the consequences (e.g. disease-related fear, threat, and anxiety) are indisputable stressors. Many studies have revealed that people presented with psychological responses (such as anxiety and depression) to the SARS and MERS outbreak in different regions (5–7). A study on online mental health surveys associated with the COVID-19 outbreak has recently been published, which targeted different populations, and revealed that the prevalence of depression and anxiety was 50.7% and 44.7%, respectively (8). In addition, patients with a history of psychiatric illnesses and who were isolated had a high risk of anxiety and anger at 4-6 months after withdrawal from isolation. The neurotransmitter abnormalities in the emotional center of the cerebrum may lead to poor control of symptoms of anxiety and anger in these patients (7, 9).

At present, many solutions have been presented to address the mental health of patients and professionals affected by the disease, which were mainly based on the notification of basic principles for emergency psychological crisis interventions for the 2019-nCoV pneumonia released by the National Health Commission of China (10). The National Health Commission guidelines have different points for different groups, including patients with confirmed or suspected COVID-19, medical staff, people who have had close contact with patients, susceptible people (e.g. children, pregnant women, and the elderly), and the general public. For example, for the general public, the most important measures were to avoid crowds, staying informed, maintaining constant communication within the family, making healthy eating and exercise a family activity, practicing good hygiene and prevention strategies, having adequate relaxation (e.g. self-care, rest, and sleep), and staying connected with extended families, friends, and basic resources. In addition, many online mental health education programs, literature on COVID-19 prevention, control and mental health education, and online psychological counselling services have been widely used during the COVID-19 outbreak. Meanwhile, a conceptual framework has been developed by early career psychiatrists across the world, which can be used to guide the development, implementation, and evaluation of mental health interventions during the ongoing COVID-19 pandemic (11). In order to ensure the safety of these students, all the students were instructed to be quarantined at home when the disease outbreak began, including the medical students. However, unlike other students, medical students have a deeper understanding of the disease, making them more anxious during the quarantine period. Apart from suffering from anxiety about COVID-19, students who were scheduled to attend classes taught by teachers from the hospital or some senior students who were scheduled to commence clinical practice within the hospital, and other students preparing for their postgraduate entrance examination were also quarantined at home. Furthermore, there was a discontinuation of the normal transport network within the city. This further disrupted the academic schedule of these students, thereby potentially making them depressed or anxious. Therefore, it was important to analyze whether there were mental health problems affecting medical students during the COVID-19 outbreak, and whether there were gender, geographic, or grade differences, if this problem exists. To further understand the physical and mental health of these medical students, an online mental health survey associated with the COVID-19 outbreak was conducted *via* the WeChat-based survey program Questionnaire Star (8), which is an instant messaging social media application that has been widely used in China and among Chinese students.

## Materials and Methods

### WeChat-Based Survey Program Questionnaire Stair: Basic Features

The questionnaire was designed by a neurologist (Qing Zhu), who has 16 years of specialist experience. It mainly comprised of demographic information and location, as well as questions from the Patient Health Questionnaire-9 (PHQ-9) and Generalized Anxiety Disorder-7 (GAD-7) scale.

### WeChat-Based Survey Program Questionnaire Stair: Recruitment Procedure

The data was collected from medical students who are presently enrolled at Tongji Medical College, Huazhong University of Science and Technology, and have been quarantined at home since the COVID-19 outbreak, from February 23, 2020 to April 2, 2020. The questionnaire was sent to the WeChat group of each class. These students were informed that the purpose of the survey was to understand the impact of the COVID-19 epidemic on the mental health of medical college students. Students who were willing to participate in the survey completed the questionnaires. And these students were instructed to write down a recent incident or occurrence that would have made them feel depressed or anxious, if this existed. The present study was approved by the Medical Ethics Committee of Tongji Medical College of Huazhong University of Science and Technology (No. 20200044). Informed consent for this retrospective study was waived.

### WeChat-Based Survey Program Questionnaire Stair: Depression Screening Tool

The depression of the participants was assessed using the 9-item Patient Health Questionnaire-9 (PHQ-9), which has been shown to be sensitive and specific enough to screen adolescents with suspected depression (12). Furthermore, the PHQ-9 is the most frequently used tool designed for screening depression in primary care (13–15), and is a 9-item depression module from the full PHQ. The participants were instructed to answer the questions in order to evaluate the frequency of the particular symptoms they felt during the quarantine period due to the Covid-19 outbreak. The answers were scored as follows: 0 point for not at all, 1 point for several days, 2 points for more than half of the days, and 3 points for nearly every day. The score was scaled from 0-27 (0-4: without depression symptoms, 5-9: with mild depression symptoms, 10-14: with moderate depression symptoms, 15-19: with moderate to severe depression symptoms, and 20-27: with severe depression symptoms) (14).

### WeChat-Based Survey Program Questionnaire Stair: Anxiety Screening Tool

Anxiety was assessed using the 7-item Generalized Anxiety Disorder-7 (GAD-7) scale, which has been used to evaluate the mental health status of people after the MERS outbreak (7). The GAD-7 scale comprises of seven highly relevant questions selected from 13 items (nine questions from the Diagnostic and Statistical Manual of Mental Disorder, 4^th^ Edition, and four questions from the Anxiety Symptom Scale). For each item, the subjects were asked about their frequency of experiencing each one of the feelings during their quarantine period for COVID-19. The answers were scored as follows: 0 point for not at all, 1 point for several days, 2 points for more than half of the days, and 3 points for nearly every day. The score was scaled from 0-21 (0-4: without anxiety symptoms, 5-9: with mild anxiety symptoms, 10-14: with moderate anxiety symptoms, and 15-21: with severe anxiety symptoms) (7).

### Statistical Analysis

All statistical analyses were performed using the IBM SPSS Statistical Software (version 19). Counting data was presented in counts (the percentage of the total). The chi-square test was used to determine whether there were significant differences between the number of asymptomatic and symptomatic students, in terms of different genders, geographical locations, and grades. The Kruskal-Wallis test was used to determine whether there were significant differences among the number of different degrees of symptomatic students, in terms of different genders, geographical locations, and grades. A *P*-value of <0.05 was considered statistically significant.

## Results

### WeChat-Based Survey Program Questionnaire Stair: Sample Characteristics

Students from six grades participated in the survey. Grades 1-5 were undergraduates, while the others were students in an eight-year program, who were in their 7^th^ year. Approximately five hundred students received the questionnaire. A total of 217 students (Grade 1: 9, Grade 2: 9, Grade 3: 85, Grade 4: 52, Grade 5: 35, and Grade 7: 27) were enrolled in the survey, which consisted of 127 (58.5%) females and 90 (41.5%) males. Among these participants, 167 (77.0%) participants were living outside Hubei province when they completed the survey. This information is presented in **Table 1**.

**Table 1:** Demographic characteristics of the participants in the survey.

	Participants (n=217)
Age(y)	21.7 ± 1.7 (18-27)
Grade	
1	9 (4.1%)
2	9 (4.1%)
3	85 (39.2%)
4	52 (24.0%)
5	35 (16.1%)
7	27 (12.5%)
**Gender**	
Male	90 (41.5%)
Female	127 (58.5%)
**Geographical position**	
Inside Hubei province	50 (23.0%)
Outside Hubei province	167 (77.0%)
**PHQ-9**	
0-4 (asymptomatic)	140 (64.5%)
5-9 (mild)	53 (24.4%)
10-14 (moderate)	22 (10.1%)
15-19 (moderate to severe)	1 (0.5%)
20-27 (severe)	1 (0.5%)
**GAD-7**	
0-4 (asymptomatic)	169 (77.9%)
5-9 (mild)	32 (14.7%)
10-14 (moderate)	14 (6.4%)
15-21 (severe)	2 (1.0%)

GAD-7, Generalized Anxiety Disorder-7; PHQ-9, Patient Health Questionnaire-9; y, year.

### Questionnaire Results

Based on the results of the questionnaire, it was found that the prevalence of depression (defined as a total score of ≥5 in the PHQ-9) was 35.5% and the prevalence of anxiety (defined as a total score of ≥5 in the GAD-7) was 22.1%. The majority of students who were in a depressed or anxiety state were mild or moderate.

For the different genders, the results of the PHQ-9 demonstrated that 50 (30.0%) of the 127 females and 27 (39.4%) of the 90 males were in a state of depression. Among the female students in a state of depression, 33 (26.0%) students had mild depression symptoms, 16 (12.6%) students had moderate depression symptoms, 1 (0.8%) student had moderate to severe depression symptoms, and 0 (0%) students had symptoms of severe depression. Among the male students in a state of depression, 20 (22.2%) students had mild depression symptoms, 6 (6.7%) students had moderate depression symptoms, 0 (0%) students had moderate to severe depression symptoms, and 1 (1.1%) student had severe depression symptoms. For the GAD-7, 30 (23.6%) of the 93 females and 18 (20.0%) of the 61 males were in a state of anxiety. Among the female students in a state of anxiety, 19 (15.0%) students had mild anxiety symptoms, 11 (8.6%) students had moderate anxiety symptoms, and 0 (0%) students had severe anxiety symptoms. Among the male students in a state of anxiety, 13 (14.5%) students had mild anxiety symptoms, 3 (3.3%) students had moderate anxiety symptoms, and 2 (2.2%) students had severe anxiety symptoms. There were no significant differences in the prevalence of depression (*P*=0.155) and anxiety (*P*=0.526) between the different genders of students (**Table 2**).

**Table 2:** The Questionnaire results of PHQ-9 and GAD-7.

	Gender			Geographical position			Grade						
	Male (n=90)	Female (n=127)	P value	Inside Hubei province (n=50)	Outside Hubei province (n=167)	P vlaue	1 (n=9)	2 (n=9)	3 (n=85)	4 (n=52)	5 (n=35)	7 (n=27)	P vlaue
**PHQ-9**			P=0.155			P=0.356							P=0.097
0-4 (asymptomatic)	63(70.0%)	77(60.6%)		35(70.0%)	105(62.9%)		7(77.8%)	5(55.6%)	50(58.8%)	39(75.0%)	26(74.3%)	13(48.1%)	
≥5(symptomatic)	27(30.0%)	50(39.4%)		15(30.0%)	62(37.1%)		2(22.2%)	4(44.4%)	35(41.2%)	13(25.0%)	9(25.7%)	14(51.9%)	
Stage			P=0.521			P=0.911							P=0.089
5-9 (mild)	20(22.2%)	33(26.0%)		10(20.0%)	43(25.7%)		0(0.0%)	4(44.4%)	24(28.2%)	11(21.2%)	4(11.4%)	10(37.0%)	
10-14 (moderate)	6(6.7%)	16(12.6%)		5(10.0%)	17(10.2%)		2(22.2%)	0(0.0%)	9(10.6%)	2(3.8%)	5(14.3%)	4(14.9%)	
15-19 (moderate to severe)	0(0.0%)	1(0.8%)		0(0.0%)	1(0.6%)		0(0.0%)	0(0.0%)	1(1.2%)	0(0.0%)	0(0.0%)	0(0.0%)	
20-27 (severe)	1(1.1%)	0(0.0%)		0(0.0%)	1(0.6%)		0(0.0%)	0(0.0%)	1(1.2%)	0(0.0%)	0(0.0%)	0(0.0%)	
**GAD-7**			P=0.526			P=0.981							P=0.220
0-4 (asymptomatic)	72(80.0%)	97(76.4%)		39(78.0%)	130(77.8%)		6(66.7%)	7(77.8%)	65(76.5%)	45(86.5%)	29(82.9%)	17(63.0%)	
≥5(symptomatic)	18(20.0%)	30(23.6%)		11(22.0%)	37(22.2%)		3(33.3%)	2(22.2%	20(23.5%)	7(13.5%)	6(17.1%)	10(37%)	
Stage			P=0.747			P=0.582							P=0.578
5-9 (mild)	13(14.5%)	19(15.0%)		8(16.0%)	24(14.4%)		1(11.1%)	1(11.1%)	15(17.6%)	6(11.6%)	4(11.4%)	5(18.5%)	
10-14 (moderate)	3(3.3%)	11(8.6%)		3(6.0%)	11(6.6%)		2(22.2%)	1(11.1%)	3(3.5%)	1(1.9%)	2(5.7%)	5(18.5%)	
15-21 (severe)	2(2.2%)	0(0.0%)		0(0.0%)	2(1.2%)		0(0.0%)	0(0.0%)	2(2.4%)	0(0.0%)	0(0.0%)	0(0.0%)	

GAD-7, Generalized Anxiety Disorder-7; PHQ-9, Patient Health Questionnaire-9.

For the different geographical locations, the PHQ-9 results revealed that 15 (30.0%) of the 50 students in Hubei province were in a state of depression, while 62 (70%) of the 167 students who were living outside of Hubei province exhibited depressive symptoms. Among the depressive students who were living in Hubei province, 10 (20%) students had mild depression symptoms, 5 (10%) students had moderate depression symptoms, 0 (0%) student had moderate to severe depression symptoms, and 0 (0%) students had severe symptoms of depression. On the other hand, among the depressed students living outside Hubei province, 43 (25.7%) students had mild depression symptoms, 17 (10.2%) students had moderate depression symptoms, 1 (0.6%) student had moderate to severe depression symptoms, and 1 (0.6%) student had symptoms of severe depression. The GAD-7 results revealed that 11 (22.0%) of the 50 students in Hubei province were in a state of anxiety, while 37 (22.2%) of the 167 students outside Hubei province had anxiety. Among the students who were in a state of anxiety in Hubei province, 8 (16.0%) students had mild anxiety symptoms, 3 (6.0%) students had moderate anxiety symptoms, and 0 (0%) students had severe anxiety symptoms. Among the students with anxiety who were living outside Hubei province, 24 (14.4%) students had mild anxiety symptoms, 11 (6.6%) students had moderate anxiety symptoms, and 2 (1.2%) students had severe anxiety symptoms. There were no significant differences in the prevalence of depression (*P*=0.356) and anxiety (*P*=0.981) between the students in Hubei province and those who were residing outside the province. Furthermore, no significant differences were found in terms of the severity of depression (*P*=0.911) and anxiety (*P*=0.582) between students in Hubei province and those outside Hubei province (**Table 2**).

With regards to the different grades, the PHQ-9 results revealed that 2 (22.2%) of the 7 students in grade 1, 4 (44.4%) of the 9 students in grade 2, 35 (41.2%) of the 85 students in grade 3, 13 (25%) of the 52 students in grade 4, 9 (25.7%) of the 35 students in grade 5, and 14 (51.9%) of the 27 students in grade 7 were in a state of depression. In addition, the GAD-7 results revealed that 3 (22.2%) of the 7 students in grade 1, 2 (44.4%) of the 9 students in grade 2, 20 (41.2%) of the 85 students in grade 3, 7 (25%) of the 52 students in grade 4, 6 (25.7%) of the 35 students in grade 5, and 10 (51.9%) of the 27 students in grade 7 were in a state of anxiety. Detailed information on the proportion of students in a state of depression and anxiety are presented in **Table 2**. There were no significant differences in the prevalence of depression (*P*=0.097) and anxiety (*P*=0.220) among students in different grades. Furthermore, no significant differences in the disease severity of depression (*P*=0.089) and anxiety (*P*=0.578) were found among students in different grades (**Table 2**).

Some students also mentioned that something made them depressed or anxious. Most of them felt upset due to the disease (COVID-19), which led them to be quarantined at home, and expressed that this situation made their life generally boring. Furthermore, some of them felt unhappy, because the quarantine meant that they could not go out in the event that they had disputes with their parents. Apart from these aforementioned reasons, worrying about the disrupted schedule of the postgraduate entrance exam also resulted in bad moods. Numerous other reasons were also stated. For example, several students were worried because they were separated from their lovers.

## Discussion

The main goal of the present study was to determine whether there were mental health problems in medical students during the COVID-19 outbreak quarantine period. According to these results, it can be concluded that approximately one-third of students who participated in the survey were in a state of mild or moderate depression, one-fifth of these students were in a state of mild or moderate anxiety, and merely one or two were severe. These results are consistent with published findings on individual adjustment during the COVID-19 epidemic in general public samples (8), even for those who were not in direct contact with the disease, illustrating that the psychiatric impact of the disease on the general population is significant. As of the end of January 2020, a nationwide quarantine was carried out, and people who were isolated in their homes were potentially apprehensive about the possibility of infection, and anxious over the high infectivity and uncertain mortality rate of COVID-19. In addition, the anxiety could spread to family members who are also concurrently isolated in their homes. Students had been under quarantine in their homes during the period of the COVID-19 outbreak, which lasted for more than three months. The prolonged time of isolation and the associated consequences might induce depression and anxiety among these students.

For students in a state of depression or anxiety, most of them were mild or moderate, and merely one or two were severe. However, there were no gender differences in the prevalence of depression and anxiety, which might suggest that both genders were equally psychologically affected by the COVID-10 epidemic. This is consistent with findings from a previous study on the SARS epidemic (16). Contrary to the conclusions from a prior study, which reported that college students whose family and university were in the same city (Beijing) demonstrated lower psychological symptoms than their peers whose families lived in other cities during the SARS outbreak (16), the present survey revealed that students in Hubei province and outside Hubei province had no significant difference in the prevalence of depression and anxiety. The possible reason for this is because only a small proportion of students in Hubei province participated in the survey. Although a greater impact may have been observed for senior students, the results of the survey revealed that there was no significant difference in the prevalence of depression and anxiety among the different grades. This may have been affected by the small number of senior students who participated in the present study.

Although the present survey mainly screened for depression and anxiety, a previous study demonstrated that the progression of negative emotions such as anxiety, which are experienced in the early stages of disasters, can lead to post-traumatic stress disorder when left without intervention. However, if early mental health care measures are implemented, such consequences can be obviated (17). It was advantageous that the majority of students who were in a depressed or anxious state had mild or moderate symptoms. This allowed for a favorable outcome when appropriate measures were taken.

Many solutions presently available in China and worldwide can be used for individuals with mental health problems. As indicated by one of the recently published articles, online mental health education using social networking sites and applications, such as WeChat, Weibo, and TikTok, has been widely used. Furthermore, literature on mental health education has been expeditiously published, and free electronic copies have been distributed to the public during the outbreak (8). Moreover, the American Psychiatry Association has also provided a great deal of guidance on their website (https://www.psychiatry.org/psychiatrists/covid-19-coronavirus). Meanwhile, a study on the experience of stressors, coping, and adjustment among college students during the 2003 SARS epidemic also provided some suggestions for university counseling services (16). Students should be instructed on where and how to obtain the resources, since excessive information has been disseminated on the internet. This may make it difficult for people to access the useful messages. Online courses could be launched, which could make the life of students more exciting, and this could also provide a platform for students to communicate with others. As for students with irregular living habits at home, encouraging them to abandon these habits is also necessary, since bad habits may also result in bad moods.

Apart from offering some platforms for the relief of students by themselves, teachers should pay more attention to students with moderate or severe depression or anxiety. Helping them solve the problems they encounter is also a way to relieve tension and anxiety. Nevertheless, if the symptoms of a student is too severe to be resolved by the teacher, professional diagnosis and treatment should be implemented.

### Limitations

The present study has several limitations. First, the present study was a cross-sectional study, and the investigation of the psychological and physical alterations of students across different periods of the COVID-19 epidemic would provide a better understanding of the psychiatric impact of the disease. Second, the PAQ-9 and GAD-7 were made through semi-structured diagnostic interviews, and all results were self-assessed, which may introduce bias through the reporter effect. Further studies should use a more professional method, when conditions permit. Third, the number of students who participated in the survey was small, and the possible reason is the voluntary nature of the survey. Hence, some students opted not to participate. Furthermore, the voluntary nature of the survey also made the follow-ups difficult to conduct (very few students were willing to fill in the questionnaire again). Finally, the present survey did not exclude students who previously suffered from mental illness.

## Conclusion

In general, students, especially college students, are a special group that should not be ignored during the quarantine period. Not only students in Hubei province, but also students outside Hubei should be given particular attention. The present study is one of the few studies that investigated the mental health conditions of medical college students during the COVID-19 epidemic. The present study implies that universities need to take measures to prevent, identify, and deal with the mental health problems of students during large-scale stressors (e.g. the outbreak of an infectious disease and natural disasters). Effective screening procedures would help to identify students who are at high risk of developing mental health problems, and effective interventions could prevent serious consequences.

## Data Availability Statement

The raw data supporting the conclusions of this article will be made available by the authors, without undue reservation, to any qualified researcher.

## Ethics Statement

This study was approved by Tongji Medical College of Huazhong University of Science and Technology medical ethics committee. All subjects were informed about the purpose of the study in accordance with Chinese legislation. As the students are isolated at home since the COVID-19, written informed consent from the participants was not required to participate in this study in accordance with the national legislation and the institutional requirements.

## Author Contributions

QZ contributed to the conception of the study. WF contributed significantly to analysis and manuscript preparation. JL and JW performed the data analyses and wrote the manuscript. JM helped modify the manuscript. CZ contributed to the interpretation and discussion of the results of the analysis.

## Funding

This study was supported by the National Natural Science Foundation of China (Grant Nos. 81701653, 81701673) and Hubei Provincial Natural Science Foundation of China (Grant Nos. 2019CFB639, 2019CFB497).

## Conflict of Interest

The authors declare that the research was conducted in the absence of any commercial or financial relationships that could be construed as a potential conflict of interest.
