# Corrigendum: Online Mental Health Survey in a Medical College in China During the COVID-19 Outbreak

**DOI:** 10.3389/fpsyt.2020.00845

**Published:** 2020-08-14

**Authors:** Jia Liu, Qing Zhu, Wenliang Fan, Joyman Makamure, Chuansheng Zheng, Jing Wang

**Affiliations:** ^1^ Department of Radiology, Union Hospital, Tongji Medical College, Huazhong University of Science and Technology, Wuhan, China; ^2^ Hubei Province Key Laboratory of Molecular Imaging, Wuhan, China; ^3^ Department of Neurology, Union Hospital, Tongji Medical College, Huazhong University of Science and Technology, Wuhan, China

**Keywords:** COVID-19, depression, anxiety, PHQ-9, GAD-7

In the published article, there was an error in affiliation 1. Instead of “Department of Radiology, Tongji Medical College, Union Hospital, Huazhong University of Science and Technology, Wuhan, China,” it should be “Department of Radiology, Union Hospital, Tongji Medical College, Huazhong University of Science and Technology, Wuhan, China.”

In addition, there was an error in affiliation 3. Instead of “Department of Neurology, Tongji Medical College, Union Hospital, Huazhong University of Science and Technology, Wuhan, China,” it should be “Department of Neurology, Union Hospital, Tongji Medical College, Huazhong University of Science and Technology, Wuhan, China.”

The authors apologize for this error and state that this does not change the scientific conclusions of the article in any way. The original article has been updated.

